# Nipah virus infection: preparedness for the pathological diagnosis of an emerging Paramyxoviridae disease with epidemic potential

**DOI:** 10.1590/S1678-9946202567040

**Published:** 2025-06-27

**Authors:** Cinthya dos Santos Cirqueira Borges, Ana Maria Gonçalves, Fernanda Alencar Rodrigues, Thais de Souza Lima, Ana Catharina Seixas Nastri, Venâncio Avancini Ferreira Alves, Mirian Nacagami Sotto, Thais Mauad, Soon Hao Tan, Paulo Hilário Nascimento Saldiva, Luiz Fernando Ferraz Silva, Kum Thong Wong, Marisa Dolhnikoff, Amaro Nunes Duarte-Neto

**Affiliations:** 1 Instituto Adolfo Lutz, São Paulo, São Paulo, Brazil; 2 Universidade de São Paulo, Faculdade de Medicina, Departamento de Patologia, São Paulo, São Paulo, Brazil; 3 Universidade de São Paulo, Faculdade de Medicina, Hospital das Clinicas, Divisão de Clínica de Moléstias Infecciosas e Parasitárias, São Paulo, São Paulo, Brazil; 4 University of Malaya, Faculty of Medicine, Department of Pathology, Kuala Lumpur, Malaysia; 5 Universidade de São Paulo, Serviço de Verificação de Óbitos da Capital, São Paulo, São Paulo, Brazil

Sao Paulo, April 3^rd^, 2025

Dear Editor,

In the current globalization scenario, with massive movements of people over long distances in short periods of time, combined with climatic and social changes, there is a fear that infectious agents of high virulence, especially viruses, or even a "Disease X" will cause a new epidemic or pandemic^1^. There is a real risk of accidental or even deliberate movement of a person/people across borders during the viremic phase, enabling the agent to be transmitted from person to person, or even by arthropods such as *Aedes* spp. present on several continents. This is a global health issue, with recent examples such as the H1N1 influenza epidemic^2^, COVID-19^3^ and Mpox^4^. The Zika epidemic in Brazil probably had a similar start back in 2015^5^.

In 2018, the World Health Organization (WHO) published a list of priorities for research and development for future epidemic preparedness - the "Blue Print" list, which was updated in 2022 - that includes SARS-CoV-2 and variants, Middle East respiratory syndrome (MERS) and Severe acute respiratory syndrome (SARS), as well as the Zika virus (ZIKV), the yellow fever virus (YFV), the Ebola virus (EBOV), the Lassa virus (LASV), the Crimean-Congo hemorrhagic fever virus (CCHFV), the Marburg virus (MARV), the Rift Valley fever virus (RVFV), the hantavirus, and the Nipah virus (NiV)^6,7^. Most institutions, research funding agencies and scientific texts aim to prepare for future epidemics, in terms of diagnosis, focus on rapid clinical tests with high sensitivity and specificity at low cost^8^.

Diagnosis is one of the main pillars of epidemic preparedness, enabling "case definition," rapid initiation of appropriate treatment, introduction of control measures and prevention of the spread of serious infectious diseases^8^. However, what happens when the first index case of an outbreak is a fatality without a positive diagnostic test before death? The answer is autopsy. The autopsy is a millennia-old medical tool, and there are many outbreaks and epidemics in medical history that have been identified by it. Autopsies are essential procedures when the diagnosis was not clinically suspected or when definitive tests were not carried out before death. It can confirm the case using techniques such as macroscopy, histopathology, electron microscopy, immunohistochemistry and molecular tests^9–14^.

In the last six years, the Department of Pathology of the Faculty of Medicine and the Death Verification Service of the Capital (Servico de Verificacao de Obitos da Capital [SVOC]), both affiliated to the University of Sao Paulo, Brazil, which performs more than 15,000 autopsies per year to determine the natural cause of death in Sao Paulo city, have played an important role in recent epidemics. During H1N1 (2009), yellow fever (2018–2019), arenavirus (2019), COVID-19 (2020–2022), mpox (2022) and dengue (2024) epidemics, autopsies were crucial not only to confirm deaths, but also to establish differential diagnoses, validate new autopsy modalities, use molecular diagnostic tests in post-mortem tissue sampling to determine viral systemic spread and viral genome sequencing, study the pathogenesis of severe diseases, including vaccine reactions, and provide medical education^2,3,4,12–14^.

Despite all these positive results in a harsh medical environment, several obstacles to having a validated diagnosis *in situ* have been observed in our institution during these epidemics. Some of these difficulties are the high cost of commercial-specific primary antibodies, a lower budget for funding research in our setting, the long period to import these reagents from abroad, bureaucratic issues, and the lack of positive controls. Although molecular diagnostics contribute substantially to the definitive diagnosis of infection, immunohistochemistry (IHC) and *in situ* hybridization are greatly important, especially when only formalin-embedded tissues are available, as well as for understanding viral infection in different cellular components of affected organs^15^.

Considering the importance of autopsies for epidemics in our environment and the obstacles that have been pointed out, as a preparatory measure for future events, the Department of Pathology of the Faculty of Medicine and the SVOC launched the PREPARE project (Preparedness in Pathology for Rapid Response in Epidemics) at the end of 2023. The project aims to acquire and standardize reagents for *in situ* diagnosis of infections by highly virulent pathogens, following the WHO Blueprint list; monitor infectious diseases in the autopsy service during seasonal periods; continue education in preparedness for possible future epidemics, including diseases identification by histopathology, and the formation of a collaborative network of laboratories to share experiences and supplies^6–8^. PREPARE will also be dedicated to validating new commercial IHC primary antibodies for the diagnosis of severe Brazilian endemic infections with epidemic potential, such as dengue and leptospirosis. This project is prospective, multidisciplinary and inclusive. It is funded by the National Council for Scientific and Technological Development (CNPQ) and was approved by the HCFMUSP-Ethical board (N° 5.835.201).

**Figure 1:**
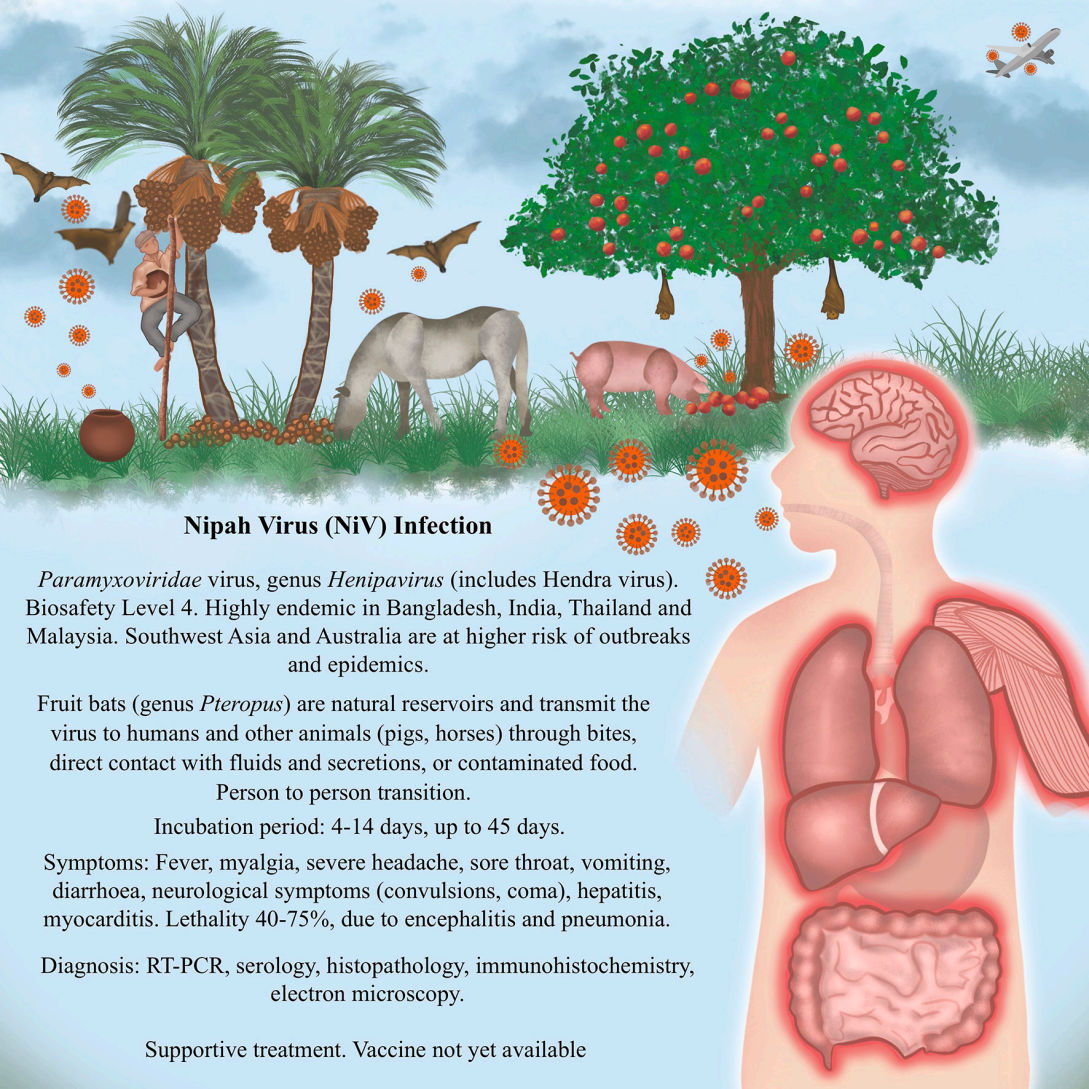
Main aspects of Nipah virus infection observed in endemic areas in India and Southeast Asia.

In collaboration with Wong *et al*.^15,16^, we started the PREPARE project with the standardization of the IHC reaction for the diagnosis of paramyxovirus infections of the genus *Henipavirus*, which includes the Nipah virus (Figure 1) and the Hendra virus, endemic to Southeast Asia and Australia, respectively. Both diseases are zoonoses of which humans are incidental hosts. In this context, the concept of One Health is highly relevant, referring to the interconnectedness of human, animal, and environmental health. As transmission of Nipah and Hendra viruses can occur from animals (such as bats or livestock) to humans, effective surveillance and control strategies require an integrated approach involving veterinary, medical, and ecological disciplines. This holistic perspective is essential for identifying sources of infection, understanding transmission dynamics, and preventing outbreaks, especially in regions where human–animal interactions are frequent. Moreover, henipaviruses represent a potential global health threat, with the capacity to cause large-scale epidemics if an infected person crosses borders during the acute phase of the disease^17^.

**Figure 2:**
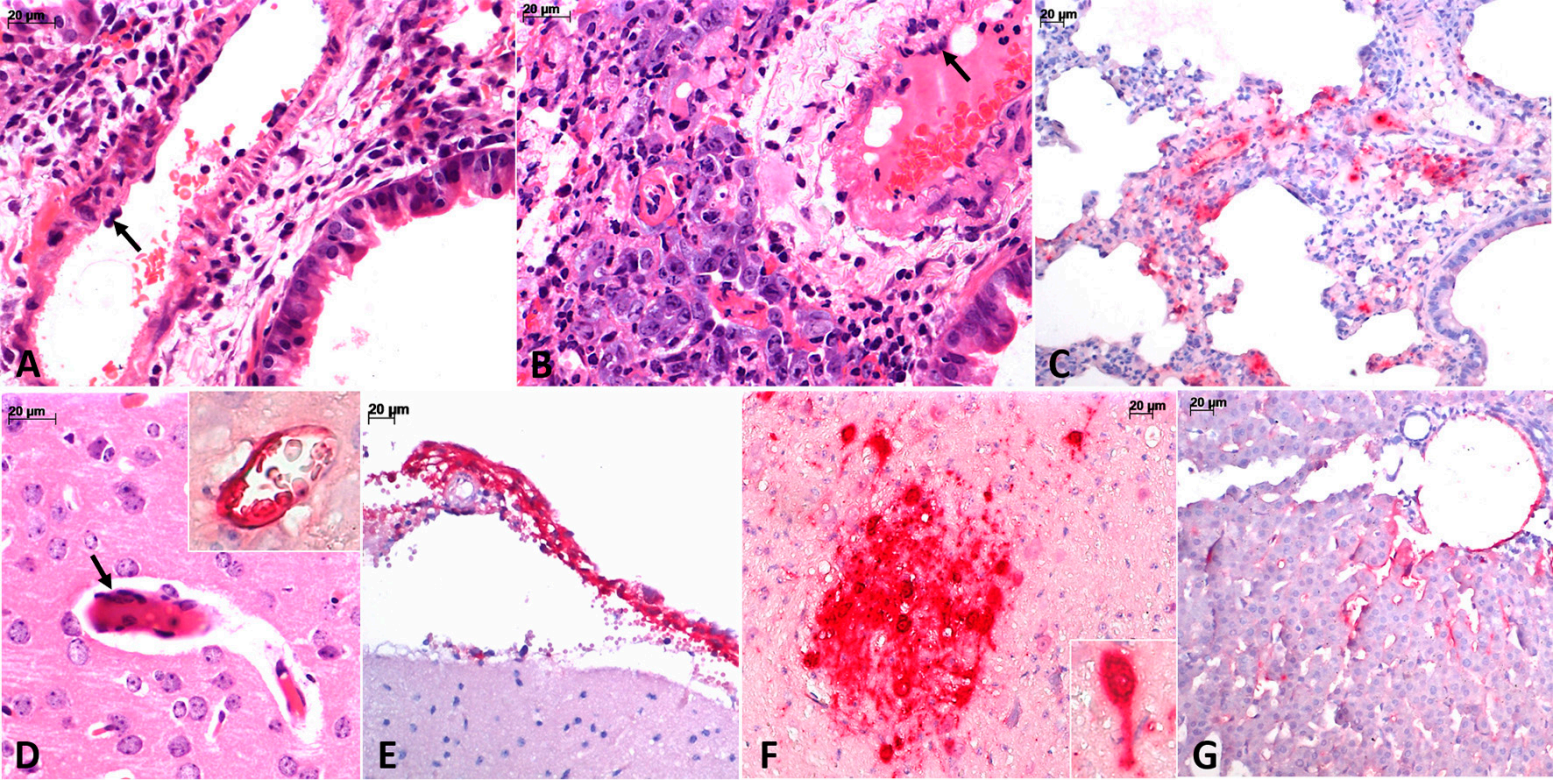
Pathological findings observed in the experimental model of *Henipavirus* (Hendra virus) infection in hamsters: (A) viral vasculitis, with inflammatory cells permeating the wall of the pulmonary artery branch, with swollen endothelial cells, some of which are multinucleated (arrow); (B) viral cytopathic effect on epithelial cells and alveolar macrophages, and endothelial cells with tumefaction and multinucleation (arrow). The inflammatory reaction is mild; (C) immunolabeling of *Henipavirus*-antigens in pulmonary endothelial and epithelial cells; (D-F) key aspects of *Henipavirus* encephalitis showing cerebral (D, inset) and meningeal (E) vessels and neurons (F) with *Henipavirus*-antigens immunolabelling. Note the multinucleated brain endothelial cells (D, arrow) and the *Henipavirus*-antigens immunolabeling in the nucleus, cytoplasm, and processes of neurons (F, inset); (G) immunolabelling of *Henipavirus*-antigens in endothelial cells of hepatic sinusoids and in Kupffer cells. H&E: A, B, D. Alkaline phosphatase: C, D (inset), E, F, G.

The Nipah virus infection has caused epidemics in Malaysia, Bangladesh, Singapore and India. On August 30, 2023, Kerala, India, experienced the largest documented outbreak of the Nipah virus disease, with 30 confirmed cases and a case fatality rate ranging from 40% to 75%^18,19^. Transmission occurred via consumption of palm sap contaminated by contact with infected fruit bats and, in approximately half of cases, via direct human-to-human contact^18,19^. The Nipah virus infection initially presents as a non-specific viral illness, but can rapidly progress to severe encephalitis, pneumonia, nephritis, and hepatitis, with high mortality rates of 40-75%^15–19^. The main histological finding (Figure 2) is infection of endothelial cells, forming multinucleated cells, with vasculitis and tissue ischemia, presenting cell debris. Lung and liver epithelial cells, cardiomyocytes, and neurons are also affected with cytopathic changes. The inflammatory response is usually mild and consists mainly of lymphocytes, histiocytes, and a few neutrophils. Reactive microglia are common. In human encephalitis cases caused by the henipavirus, the most severe form of the disease, the neuronal body and axon may show eosinophilic viral inclusions very similar to the Negri bodies seen in rabies encephalitis, which may hinder precise diagnosis in Brazil^20^.

For IHC standardization, deparaffinized 3 μm-cut histological sections were subjected to heat-induced epitope retrieval with antigen retrieval solution (EDTA Decloaker, 5X– Biocare Medical; SKU:CB917) for exposure of antigenic epitopes, followed by endogenous peroxidase blocking (H2O2; 20V). Primary antibody anti-Nipah virus Nucleoprotein (monoclonal antibody 1 μL/mL, clone HL1436, code ab308369, Abcam, San Francisco, CA, USA) was incubated overnight (16 h) at 8 °C. The dilution buffer was bovine serum albumin. The signal was amplified with Mouse/Rabbit PolyDetector AP (BSB SB; BSB-0290; Santa Barbara, CA, USA) and visualized with PolyDetector ALK Magenta (BSB SB; BSB-0080; Santa Barbara, CA, USA) followed by counterstaining with Harri's hematoxylin. The anti-Nipah virus antibody showed similar performance to antigenic retrieval at low and high pH and positivity at dilutions between 1:200 and 1:5000, with the optimal dilution at 1:3000 (Figure 2). Two blocks of formalin-fixed paraffin-embedded tissues from Hendra virus-infected hamsters containing brain, lung and liver samples were examined. Positive immunolabeling was observed in all fragments, epithelial ciliary and alveolar cells, neurons and endothelial cells (meninges, brain, lung and liver) with viral cytopathic changes. Viral inclusions were used as an internal positive control. Staining intensity was graded semiquantitatively from 0 to 3+, with most positive samples showing moderate to strong (2+ to 3+) reactivity (Figure 2). This anti-Nipah virus antibody yielded negative results in external negative controls, such as non-infected tissues from patients with cardiovascular disease and in the absence of the primary antibody. It did not cross-react with measles virus (MeV), rabies virus (RABV), herpes virus (HSV) 1 and 2, cytomegalovirus (CMV), Epstein-Barr virus (EBV), ZIKV, YFV, dengue virus (DENV), chikungunya (CHYKV), mpox (MPXV), enterovirus (EV), influenza A and B (IAV, IBV), H1N1, respiratory syncytial virus (RSV), and SARS-CoV-2 cases from our autopsy archive. The IHC reactions were performed in a laboratory at 21 °C (room temperature). This IHC reaction will be available free of charge to our public reference laboratories for testing in the event of a suspected case in our region.

With the frequent global emergence/re-emergence of infectious diseases (ID), pathology and autopsy are essential in the preparedness for future epidemics^8,14^. This is likely to be important for the future of pathology specialties in the upcoming decades. To fulfill this ideal and expectation, some actions and incentives are needed: education, research programs, and grants dedicated to ID pathology and autopsy, the engagement of the medical diagnostics industry to develop specific reagents for *in situ* diagnostics, facilitating their acquisition and delivery, especially in low-income regions, and the development of a global collaborative network to share experiences and laboratory supplies (e.g., reaction controls) in the field of ID/autopsy pathology^14^.

As a conclusion, henipaviruses cause severe infections with high mortality and have the potential to cause epidemics. Therefore, preparation in histopathology is worthwhile, through knowledge of the cardinal histological findings of each disease and the standardization of *in situ* diagnosis, using rapid, widely available and low-cost methods, such as immunohistochemistry. To this end, cooperative networks among international laboratories are necessary in order to share FFPE (Formalin-Fixed Paraffin-Embedded) samples for controls and expertise. Prospects for optimizing the use of FFPE samples for laboratory preparedness to deal with possible epidemics include the study of the ultrastructural morphology of viruses, the validation of synthetic controls for polymerase chain reaction (PCR) tests, and next-generation sequencing (NGS).
