# ERRATUM

**DOI:** 10.1590/S1678-9946202567040err

**Published:** 2025-08-25

On the Letter to the Editor “Nipah virus infection: preparedness for the pathological diagnosis of an emerging Paramyxoviridae disease with epidemic potential”, published in the Revista do Instituto de Medicina Tropical de São Paulo, Vol. 67, e40, p. 2025 (http://doi.org/10.1590/S1678-9946202567040):

On page 1, where it reads Original Article should be read Letter to the Editor

